# Clinical Outcome Improvement in Open-Wedge High Tibial Osteotomy Using the Smartphone Application mymobility®: A Case Report

**DOI:** 10.7759/cureus.98332

**Published:** 2025-12-02

**Authors:** Toshiki Azuma, Kenichi Goshima, Ryosuke Hasama, Kayo Oari, Kentaro Sasaki

**Affiliations:** 1 Department of Rehabilitation Medicine, Kinjo University, Hakusan, JPN; 2 Department of Rehabilitation Medicine, Kanazawa Munehiro Hospital, Kanazawa, JPN; 3 Department of Orthopaedic Surgery, Kanazawa Munehiro Hospital, Kanazawa, JPN; 4 Department of Physical Medicine and Rehabilitation, Kinjo University, Hakusan, JPN

**Keywords:** high tibial osteotomy, knee osteoarthritis (koa), mymobility, pain, pain neuroscience education (pne), patient education, patient-reported outcome measures (proms), physical activity (pa), rehabilitation, smartphone applications

## Abstract

Clinical outcomes following open-wedge high tibial osteotomy (OWHTO) are influenced by factors such as preoperative varus alignment, catastrophic thinking, metabolic syndrome-related diseases, and older age. This report evaluated the effectiveness of mymobility® (Zimmer Biomet, Warsaw, IN, USA), a smartphone application, for patient education and exercise guidance in improving clinical outcomes and catastrophic thinking in a patient exhibiting these risk factors. An 83-year-old man with Kellgren-Lawrence grade 3 knee osteoarthritis underwent OWHTO. Preoperative assessments revealed a Knee Injury and Osteoarthritis Outcome Score (KOOS) of 35 for symptoms, 36 for pain, 45 for activities of daily living (ADL), 10 for sports, and 0 for quality of life (QOL); an Oxford Knee Score (OKS) of 17; and a Pain Catastrophizing Scale (PCS) score of 52, indicating poor clinical outcomes and catastrophic thinking. His daily step count, walking speed, and walking pain on the visual analog scale (VAS) were 849 steps/day, 0.8 m/s, and 50 mm, respectively. mymobility® was used for video-based exercise guidance, compliance monitoring, and chat-based anxiety management. By postoperative day 90, his step count exceeded preoperative levels, reaching 1500-2000 steps/day by day 365. His VAS score was 10 mm, and his walking speed was 1.0 m/s. He had a KOOS of 71 for symptoms, 67 for pain, 72 for ADL, 45 for sports, and 25 for QOL; an OKS of 30; and a PCS score of 17. mymobility®-based patient education and exercise guidance suggested that mymobility® effectively improved clinical outcomes and catastrophic thinking in a patient who underwent OWHTO and was presenting with older age, catastrophic thinking, and metabolic syndrome-related diseases.

## Introduction

Open-wedge high tibial osteotomy (OWHTO) is a surgical procedure for treating medial compartment knee osteoarthritis (KOA) [1]. It is indicated for patients ranging from young to older ones, many of whom desire an early return to work or sports and recreational activities [2]. Gradual increases in physical activity are crucial for early recovery, contributing to the prevention of sarcopenia [3] and improvement of postoperative clinical outcomes [4]. Wearable devices and smartphone applications, such as mymobility® (Zimmer Biomet, Warsaw, IN, USA), facilitate the monitoring of step counts, patient education via chat features, and video-based exercise, which simplify activity enhancement [5]. A study revealed that the use of mymobility® increases activity levels in patients undergoing total hip arthroplasty or knee arthroplasty, with higher activity correlating with better clinical outcomes [4]. However, its effectiveness in patients undergoing OWHTO remains unreported, warranting further investigation.

Severe pain [6], limited knee range of motion (ROM) [7], and quadriceps weakness [7] influence postoperative patient satisfaction and clinical outcomes. In OWHTO rehabilitation, preventing delayed hinge fractures requires cautious, staged weight-bearing and increases in activity, often delaying preoperative function recovery by approximately six months [8].

The use of the smartphone application mymobility® has been found to be associated with increased step counts by three months post-OWHTO, shortening the recovery time [5]. However, patients with anxiety or catastrophic thinking tend to favor extended rest [9], and those with metabolic syndrome-related diseases may avoid exercise, leading to low activity and suboptimal outcomes [10]. The chat feature, exercise guidance, and patient education offered by mymobility® could address these challenges by monitoring patient status throughout the day.

Generally, older individuals with severe knee joint pain often choose total knee arthroplasty (TKA). However, in the present case, the patient chose OWHTO due to his own strong desire to resume farm work. We believe this report is significant because in recent years, there has been a substantial increase in the number of older people wishing to return to activities. This report describes the case of an old man who presented with preoperative pain, catastrophic thinking, and metabolic syndrome-related diseases, which resulted in low physical activity. Multidisciplinary rehabilitation using mymobility® for patient education and exercise guidance increased physical activity levels, surpassing preoperative levels by three months, accompanied by substantial improvements in clinical outcomes and catastrophic thinking. These findings support the utility of mymobility® in patients undergoing OWHTO.

## Case presentation

**Figure 1:**
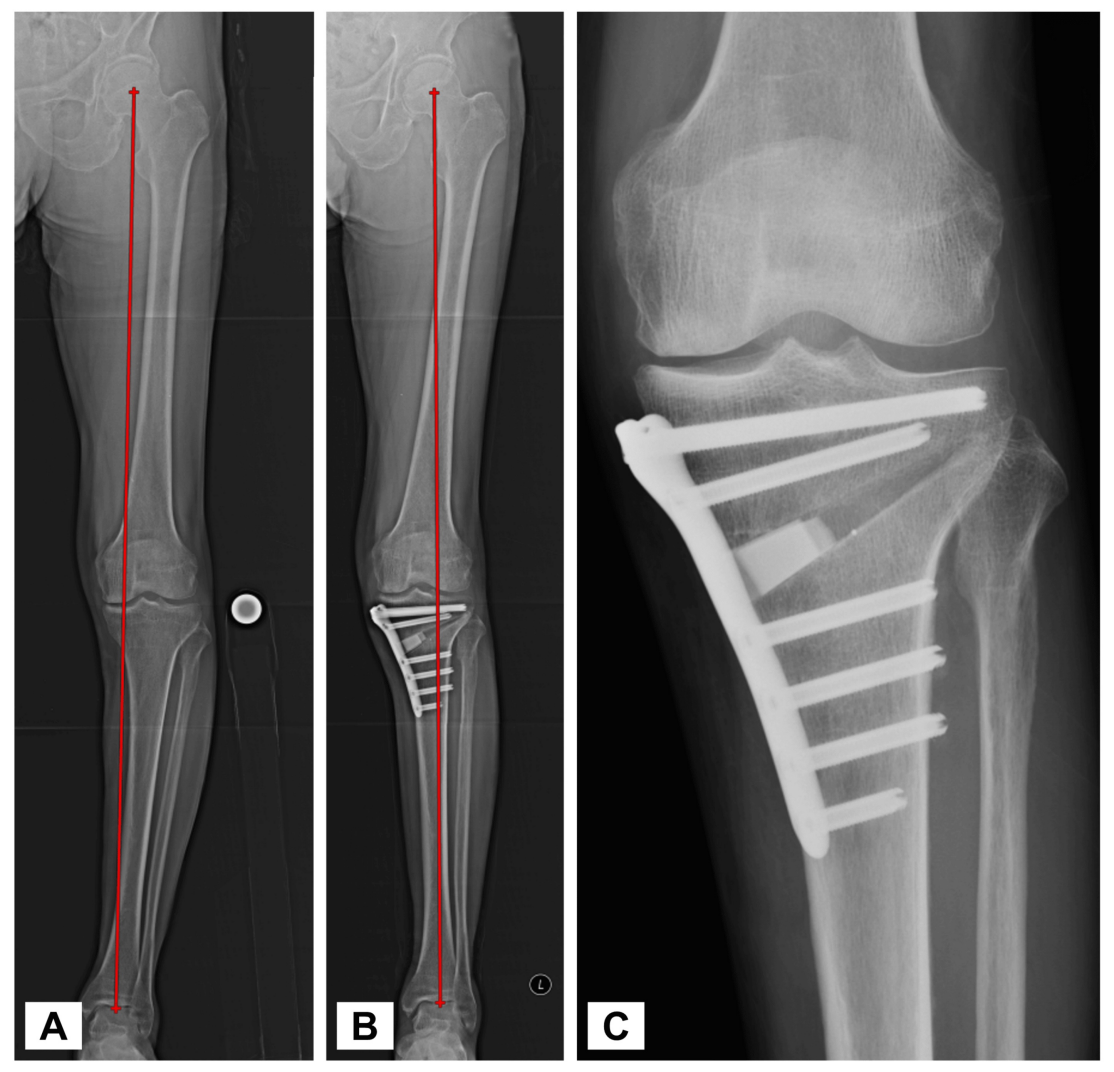
Radiographic data Full-length standing radiograph revealed that the preoperative HKA angle was 7.1° varus and the postoperative HKA angle was 3.8° valgus. (A) This is a simple X-ray image of the entire lower limb alignment in standing position before surgery. (B) This is a simple X-ray image of the entire lower limb alignment in standing position after surgery. (C) This is a simple X-ray image of the knee joint after surgery. Artificial bone was inserted into the proximal medial part of the tibia and fixed with a locking plate. HKA: hip-knee-ankle

**Figure 2:**
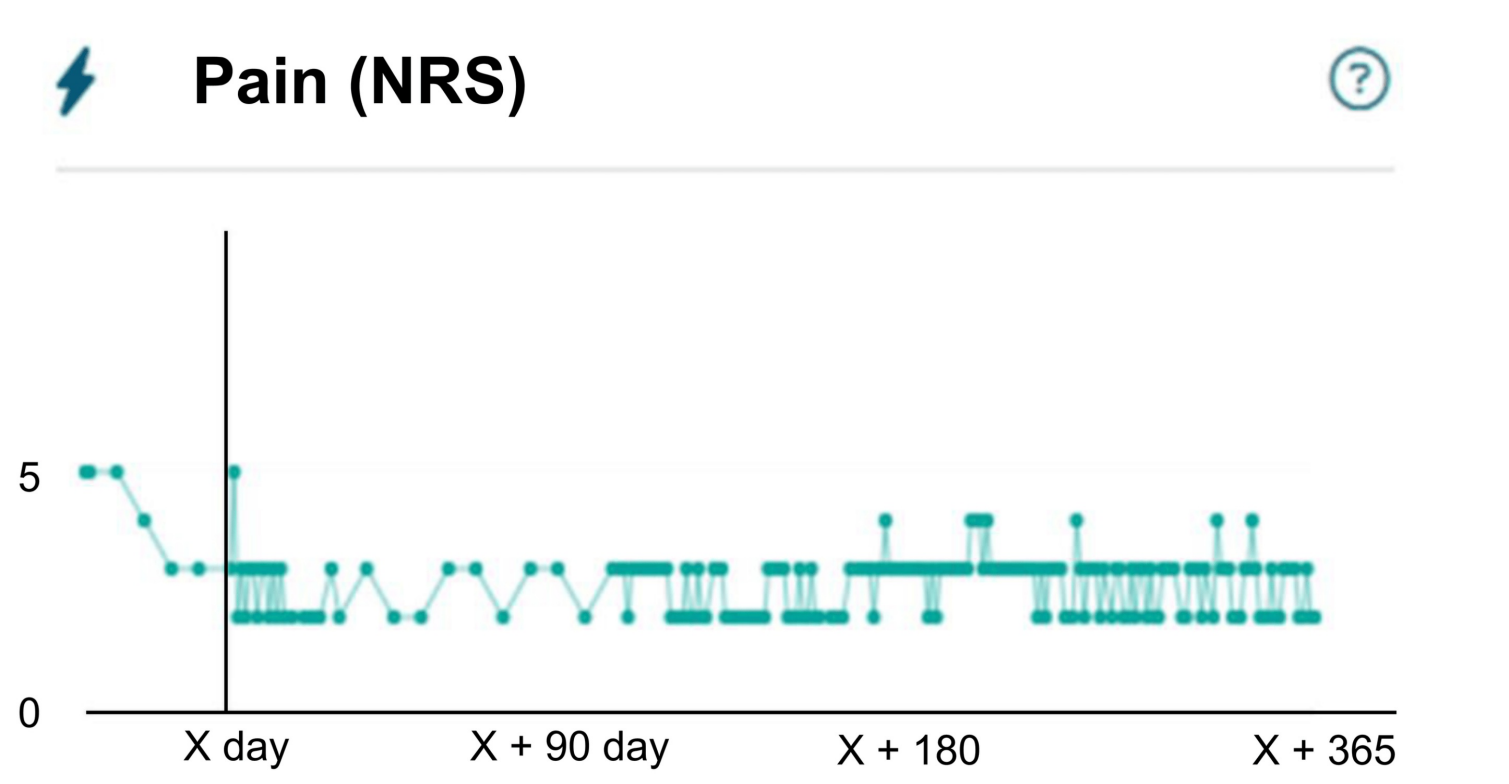
Changes in pain during gait Graph illustrating reductions in pain during walking measured using the VAS from the preoperative score of 50 mm to 30 mm on postoperative day 90 and 10 mm on postoperative day 365 in a patient undergoing open-wedge high tibial osteotomy. VAS: visual analog scale; NRS: Numerical Rating Scale

**Figure 3:**
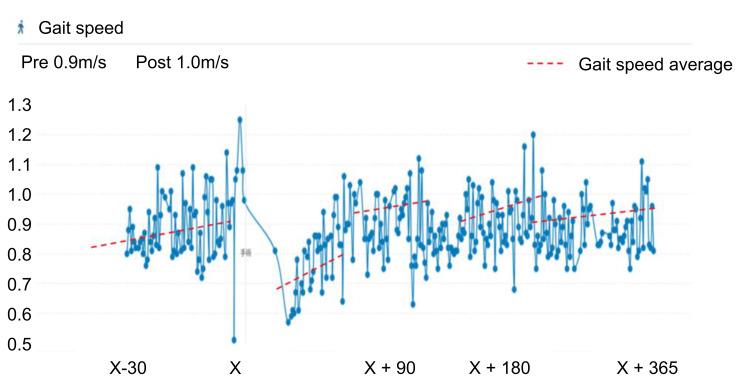
Changes in gait speed Graph depicting improvements in walking speed (m/s) from the preoperative speed of 0.8 m/s to 0.9 m/s on postoperative day 90 and 1.0 m/s on postoperative day 365 in a patient undergoing open-wedge high tibial osteotomy.

**Figure 4:**
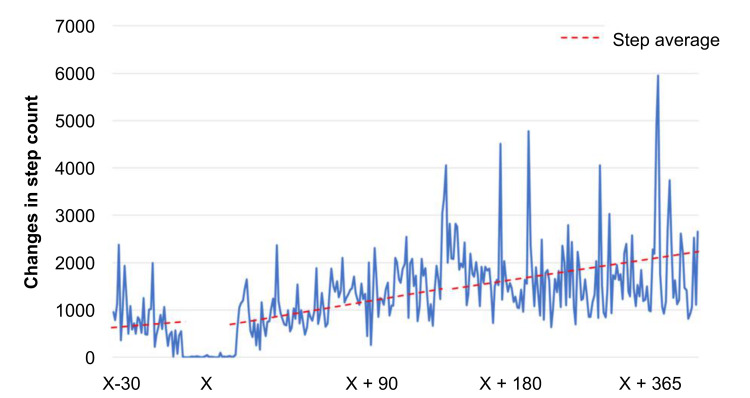
Changes in step count Graph depicting the increase in the daily step count from the preoperative step count of 849 steps/day to 1000 steps/day on postoperative day 90 and 2000 steps/day on postoperative day 365 in a patient undergoing open-wedge high tibial osteotomy.

The patient was an 83-year-old man with a body mass index of 20 kg/m^2^. He had no cognitive impairment and had comorbidities of dyslipidemia and hypertension. He lived with his wife and enjoyed gardening. Full-length standing radiographs confirmed medial KOA (Kellgren-Lawrence grade 3) with a hip-knee-ankle angle of 7.1° varus (Figure 1). Arthroscopic findings revealed a degenerative tear of the medial meniscus (from the middle to the posterior segment), treated with partial meniscectomy. The primary complaint was medial knee pain during walking and stair climbing, with a visual analog scale (VAS) score [11] of 50 mm (Figure 2). His preoperative Knee Injury and Osteoarthritis Outcome Score (KOOS) were 35 for symptoms, 36 for pain, 45 for activities of daily living (ADL), 10 for sports, and 0 for quality of life (QOL) [12]. He had an Oxford Knee Score (OKS) [13] of 17 and a Pain Catastrophizing Scale (PCS) score [14] of 52, indicating poor clinical outcomes and catastrophic thinking. At the start of using mymobility®, his preoperative walking speed and step count (median over one week) were 0.9 m/s (Figure 3) and 849 steps/day (Figure 4), reflecting low activity and reduced walking speed, respectively [5]. By postoperative day 90, his medial knee pain during walking and stair climbing improved to a VAS score of 30 mm, and independent daily living was achieved. His KOOS improved to 61 for symptoms, 61 for pain, 66 for ADL, 25 for sports, and 19 for QOL; OKS to 28; and PCS score to 17, indicating improved clinical outcomes and reduced catastrophic thinking. The step count increased to 1000 steps/day, and walking speed reached 0.9 m/s. By day 365, his VAS score was 10 mm, and his walking speed remained at 1.0 m/s. Moreover, his KOOS were 71 for symptoms, 67 for pain, 72 for ADL, 45 for sports, and 25 for QOL. His OKS and PCS scores were 30 and 17, respectively.

Surgical procedure

OWHTO is indicated for active patients with medial KOA or femoral condyle osteonecrosis, without age restrictions. The inclusion criteria were as follows: ROM extension ≥−5°, flexion ≥130°, absence of significant lateral tibiofemoral or patellofemoral joint osteoarthritis, and femorotibial angle <182°. The target postoperative weight-bearing line ratio was planned preoperatively to be between 65% and 70%. The surgical procedure was performed according to the technique described by Staubli and Jacob [15], with the osteotomy gap filled with artificial bone (AviOS, HOYA Technosurgical Corporation, Shinjuku, Japan) and fixed using an ASPIC plate® (AUSPICIOUS, Tokyo, Japan). Postoperative rehabilitation began with partial weight-bearing at one week and full weight-bearing at four weeks.

Postoperative rehabilitation

Drain removal and rehabilitation started the day following OWHTO. A physical therapist guided bedside knee ROM exercises and quadriceps isometric contractions. Video-based exercise therapy was initiated using mymobility®, including patient-performed knee flexion-extension, ankle dorsiflexion-plantarflexion, quadriceps isometric contractions, patellar mobilization, and side-lying gluteus medius resistance training. Progressive weight-bearing included half-weight-bearing with parallel bars at week 2, crutch walking at week 3, T-cane walking at week 4, and independent walking at week 5. From week 6, half-squats (knee flexion 0-90°) were performed under the guidance of a physical therapist and reinforced with mymobility® videos. The patient was discharged at week 6, and he could use a T-cane or walk independently. Outpatient rehabilitation continued three times weekly for three months, followed by weekly sessions thereafter. No complications, such as delayed postoperative tibial hinge fractures or infections, were observed 365 days after surgery. Furthermore, no postoperative loss of correction was observed.

**Figure 5:**
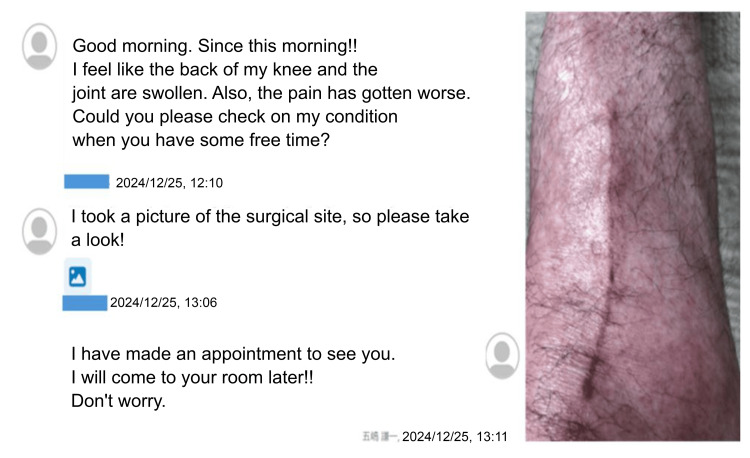
Patient education and communication using mymobility® Screenshot of the mymobility® chat feature showing the patient's communication regarding surgical site swelling and redness, with responses from the attending physician.

**Figure 6:**
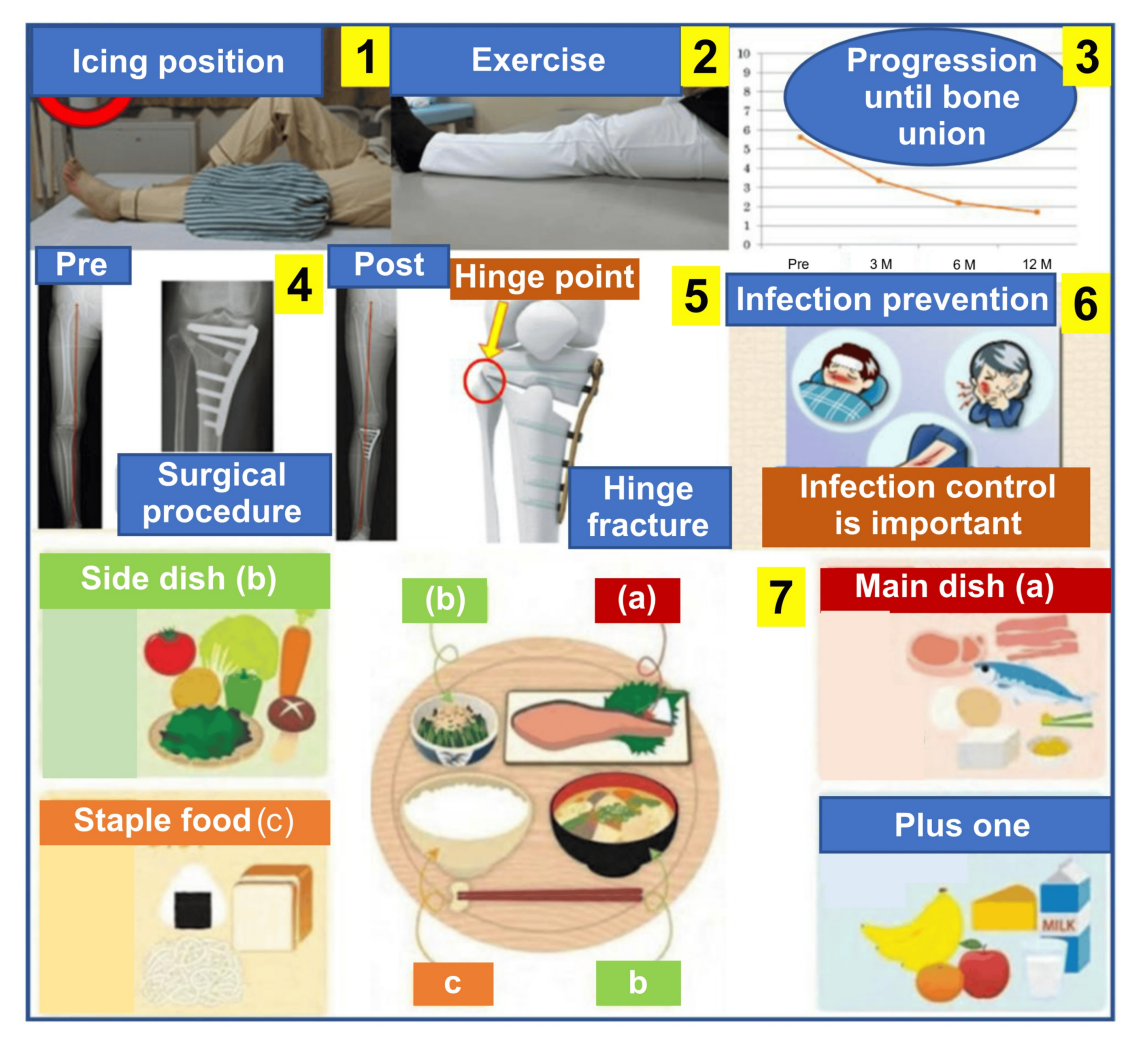
mymobility® application contents The numbers in the boxes indicate the number of each content: (1) icing method, (2) knee extension range of motion exercise, (3) patient education regarding pain, (4) explanation of surgery, (5), complications, (6) prevention of infection, and (7) dietary advice for weight loss.

Patient education and communication using mymobility®

mymobility® rehabilitation included exercise therapy, patient education, and communication (Figure 5). Preoperative hospital support from nurses and physical therapists facilitated the installation of the smartphone application. To ensure comprehension, exercise therapy was performed during outpatient rehabilitation. Patient education encompassed topics on KOA pathology, OWHTO procedures, lifestyle recommendations, and the significance of increasing activity levels, promoting self-learning (Figure 6). Daily anxieties were alleviated through face-to-face physical therapy sessions and the chat feature on mymobility®. These interventions, introduced preoperatively, enhanced the understanding of the procedure and encouraged early postoperative activity. Postoperative concerns, such as surgical site redness photographed by the patient, were evaluated by the attending physician through the chat feature. Healthcare providers, including physicians, nurses, and physical therapists, assessed pain related to the osteotomy site or muscles to alleviate anxiety and prevent catastrophic thinking. Compliance with daily exercise was monitored, with therapists or nurses encouraging adherence on non-compliant days. These supportive measures continued after discharge.

## Discussion

As presented in this report, multidisciplinary mymobility®-based patient education and exercise guidance improved activity levels and catastrophic thinking by three months, leading to enhanced clinical outcomes in a patient who underwent OWHTO and presented with advanced age, catastrophic thinking, and metabolic syndrome-related diseases. This case presents the utility of mymobility® in OWHTO rehabilitation.

Clinical outcome improvements were assessed using KOOS and OKS, validated for patients who have undergone OWHTO. Jacquet et al. reported the minimal clinically important difference (MCID) for two-year postoperative KOOS as 15.4 for pain, 15.1 for symptoms, 17 for ADL, 11.2 for sports, and 16.5 for QOL [12]. Despite the short-term follow-up (three months), the patient achieved KOOS improvements of 25 for pain, 26 for symptoms, 21 for ADL, 15 for sports, and 19 for QOL, exceeding the MCID in all domains. For OKS, Khow et al. reported MCIDs of 4.9 for patient satisfaction and 10.5 for revision in TKA [13]. The OKS improvement of 11 points exceeded these thresholds. Previous studies have suggested further improvements up to two years post-OWHTO [8]. Although the MCID of PCS is unreported, a score of <30 indicates the absence of catastrophic thinking [14]. The patient's PCS score of 17 at follow-up confirms improvement. These findings support significant clinical and psychological improvements. Notably, many of these previous studies were conducted on patients with TKA and may not all be applicable to OWHTO patients.

Postoperative clinical outcomes are linked to medial compartment decompression caused by alignment correction [1]. However, catastrophic thinking can exacerbate pain perception despite mechanical stress reduction, as the central nervous system amplifies peripheral stimuli [14]. Patients with KOA often experience chronic pain and have high PCS scores [16]. Although PCS score improvements in OWHTO are unreported, results for TKA are inconsistent [17,18]. Patient education, exercise therapy, and activity increases generally improve PCS scores [15]. In this case, preoperative mymobility®-guided exercise, combined with multidisciplinary education by physicians, physical therapists, and nurses, alleviated surgical anxiety and promoted engagement in activity, increasing the PCS scores. Preoperative resistance training was found to be effective for patients who have undergone OWHTO [19]; however, the role of patient education is less clear. The efficacy of using mymobility® in hip and knee arthroplasty education and activity support [4] likely extends to OWHTO, as demonstrated in this case. Limitations of this study are that it is a single-case report and a smartphone application was used in addition to regular rehabilitation. Thus, it cannot be said that this is the sole effect of the smartphone application. In the future, it is necessary to increase the number of cases and compare a group that received regular rehabilitation with a group that used a smartphone application in addition to regular rehabilitation.

In this case, the patient's preoperative step count of 849 steps/day increased to 1000 steps/day by day 90 and 2000 steps/day by day 365. As an 83-year-old patient with hypertension and dyslipidemia, the patient was at a high risk for cardiovascular diseases. Metabolic syndrome-related diseases showed an association with poorer one-year outcomes post-OWHTO [10]. Sustained exercise and step count increases likely improve lifestyle habits, contributing to improved clinical outcomes. An increase to 1000 steps/day from baseline reduces mortality by 23% and cardiovascular disease risk by 21% [20], indicating potential long-term benefits.

However, this single-case report lacks a sufficient sample to definitively establish the efficacy of mymobility® in OWHTO rehabilitation, requiring larger studies and statistical analyses. The patient's intact cognitive function and smartphone proficiency may not generalize to older patients or those with mild cognitive impairment. The goal of returning to moderate activity (gardening) was achieved; however, the effectiveness of mymobility® for high-intensity sports remains untested. Finally, this study addressed mild-to-moderate KOA treated with OWHTO; therefore, its applicability to severe KOA, hybrid closed-wedge high tibial osteotomy, reverse V osteotomy, or distal femoral osteotomy must be investigated further.

## Conclusions

This report describes an old man who presented with preoperative pain, catastrophic thinking, and metabolic syndrome-related diseases, resulting in low activity levels. Multidisciplinary rehabilitation using mymobility® for patient education and exercise guidance helped the patient achieve activity levels exceeding preoperative values within three months, along with notable improvements in clinical outcomes and reductions in catastrophic thinking. These findings support the effectiveness of mymobility® in the rehabilitation of patients undergoing OWHTO.
